# Classifying Tumor Reportability Status From Unstructured Electronic Pathology Reports Using Language Models in a Population-Based Cancer Registry Setting

**DOI:** 10.1200/CCI.24.00110

**Published:** 2024-11-19

**Authors:** Lovedeep Gondara, Jonathan Simkin, Gregory Arbour, Shebnum Devji, Raymond Ng

**Affiliations:** ^1^ British Columbia Cancer Registry, Provincial Health Services Authority, Vancouver, Canada; ^2^ School of Population and Public Health, University of British Columbia, Vancouver, Canada; ^3^ Data Science Institute, University of British Columbia, Vancouver, Canada

## Abstract

**PURPOSE:**

Population-based cancer registries (PBCRs) collect data on all new cancer diagnoses in a defined population. Data are sourced from pathology reports, and the PBCRs rely on manual and rule-based solutions. This study presents a state-of-the-art natural language processing (NLP) pipeline, built by fine-tuning pretrained language models (LMs). The pipeline is deployed at the British Columbia Cancer Registry (BCCR) to detect reportable tumors from a population-based feed of electronic pathology.

**METHODS:**

We fine-tune two publicly available LMs, GatorTron and BlueBERT, which are pretrained on clinical text. Fine-tuning is done using BCCR's pathology reports. For the final decision making, we combine both models' output using an OR approach. The fine-tuning data set consisted of 40,000 reports from the diagnosis year of 2021, and the test data sets consisted of 10,000 reports from the diagnosis year 2021, 20,000 reports from diagnosis year 2022, and 400 reports from diagnosis year 2023.

**RESULTS:**

The retrospective evaluation of our proposed approach showed boosted reportable accuracy, maintaining the true reportable threshold of 98%.

**CONCLUSION:**

Disadvantages of rule-based NLP in cancer surveillance include manual effort in rule design and sensitivity to language change. Deep learning approaches demonstrate superior performance in classification. PBCRs distinguish reportability status of incoming electronic cancer pathology reports. Deep learning methods provide significant advantages over rule-based NLP.

## INTRODUCTION

Cancer is a leading cause of death in every country in the world.^1^ In 2020, the International Agency for Research on Cancer estimated 10 million cancer deaths and 19.3 million new cancer cases.^1^ Cancer is a reportable disease, and the primary function of a population-based cancer registry (PBCR) is to record and collect data on all new cases of cancer in a defined population.^2^ Data from PBCRs are used to support cancer surveillance statistics, cancer control and prevention programming, and clinical research.^2^ PBCRs receive information from multiple sources to identify all new cases in a defined population.^2^ Data are primarily sourced from pathology laboratories (over 90% of reportable tumors), but PBCRs also leverage hospital records and vital statistics.^2,3^

**CONTEXT**

**Key Objective**
Can we identify tumor reportability status from pathology reports using language models (LMs)?
**Knowledge Generated**
Our proposed approach using an ensemble of LMs can successfully identify 98% of true reportable tumor pathology reports, leading to significant operational efficiency gains and aiding in timely data collection.
**Relevance *(J.L. Warner)***
Replacing tedious manual or rule-based natural language processing processes to identify reportable cases could substantially speed up registry operations. Such approaches could also decrease the lag in reporting that affects most central cancer registry operations.*
*Relevance section written by *JCO Clinical Cancer Informatics* Editor-in-Chief Jeremy L. Warner, MD, MS, FAMIA, FASCO.

Most PBCRs rely on manual record abstraction and data entry. This process is time-consuming and limits the volume and type of information that can be extracted,^4,5^ leading to delayed reporting, where complete data are often not available until 24 months or greater after a diagnosis.^5^ Increasing availability of electronic information systems are presenting new opportunities for PBCRs. Canada and the United States have shown advances in electronic reporting directly from pathology laboratories, improving case ascertainment and timeliness.^6,7^

To support processing of electronic pathology reporting, almost all PBCRs in the United States and a few in Canada use the Electronic Mapping, Reporting, and Coding (eMaRC) Plus software, developed by the US Centers for Disease Control and Prevention.^3,8^ eMaRC analyzes electronic pathology reports in Health Level Seven (HL7) format, which consists of unstructured text containing patient and facility information and diagnosis findings, among other information. eMaRC uses rule-based text analytics to identify pathology reports corresponding to reportable tumors and auto-codes key cancer registry elements from unstructured text.^3^ Rule-based approaches are known to be limited in dealing with clinical sublanguage such as nonstandard abbreviations and grammatical nuances.^4,5^

Models from deep learning, such as the ones based on transformers,^9^ have advanced the state-of-the-art in natural language processing (NLP). This presents new opportunities for cancer registries to automate data extraction from cancer pathology reports.^3-5,10-14^ This area of research combining deep learning–based NLP and cancer pathology is still in its infancy, where most studies remain a proof-of-concept and there are limited, if any, examples of operationally deployed NLP pipelines in cancer registries.^4^

In this study, we demonstrate an NLP pipeline deployed at the British Columbia Cancer Registry (BCCR), a Canadian provincial PBCR, to support the detection of reportable tumors from a real-time feed of electronic pathology. The BCCR receives all provincial pathology reports as HL7s and uses eMaRC to identify reportable tumors. However, eMaRC's rule-based approach is not robust for classifying nonreportable cancers accurately (eg, nonmelanoma skin cancers). This results in a large volume of false-positive labels (ie, nonreportable tumors classified as reportable), complicating downstream registry processes and requiring extensive manual review.

We have developed an NLP pipeline using an ensemble of language models (LMs) that analyze post-eMaRC electronic pathology reports to minimize false-positive labels. Our pipeline consists of two LMs, BlueBERT^15^ and GatorTron,^16^ both fine-tuned on BCCR's data. Different model architectures, pretraining data sets, training methodologies, fine-tuning hyperparameters, and data preprocessing methods ensure output variability for both models, aiding the ensemble to perform better than any individual model.

## METHODS

This section provides details on the BCCR, the electronic pathology reporting system, and the NLP models used for this study.

### BCCR

**FIG 1.**
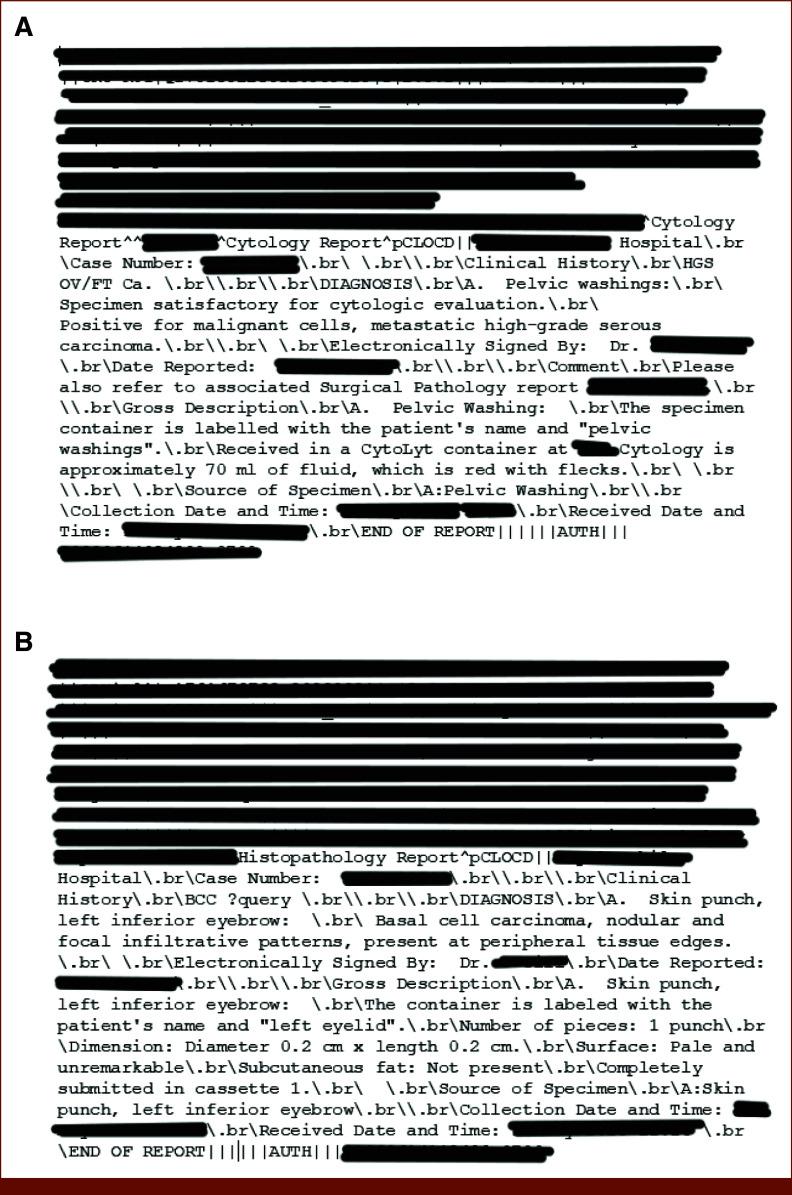
Examples of (A) reportable and (B) nonreportable cancer pathology reports. For reportable, the mention of high-grade carcinoma associated with the site (pelvis) makes it reportable; for the nonreportable, the mention of basal cell carcinoma and the site (eyebrow) makes it nonreportable.

The BCCR collects a population-based data set containing tumor information on all reportable tumors among residents of British Columbia. Nearly 90% of tumors in the BCCR are confirmed by a pathology report. BCCR conforms to national and international standards for the classification of cancer. The scope of reportability is defined by the Canadian Cancer Registry.^17^ Figure 1 shows examples of reportable and nonreportable pathology reports (HL7 format), with further details provided in the Data Supplement.

### The Provincial Electronic Pathology Feed

**FIG 2.**
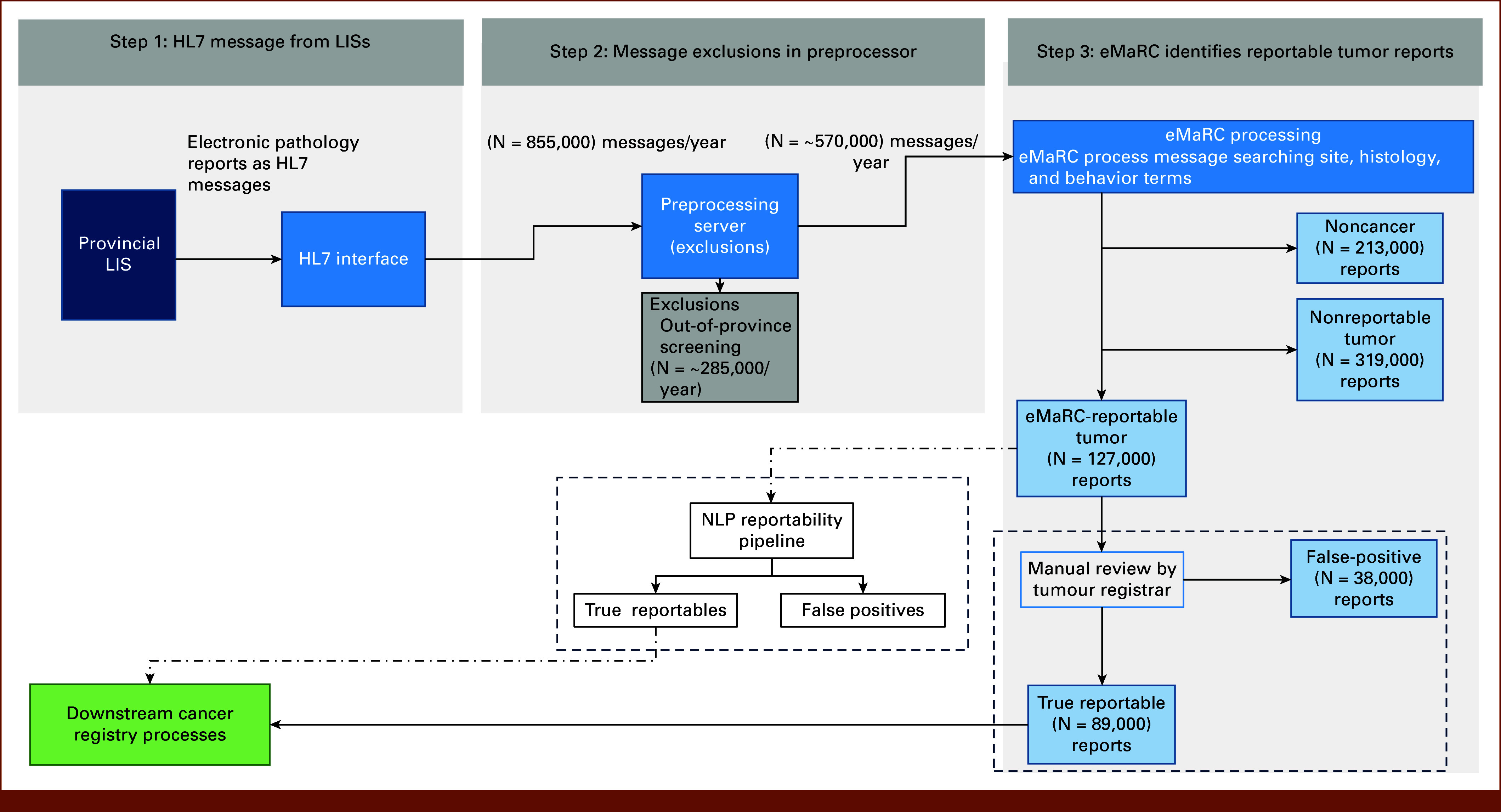
Flow of electronic pathology reports from the provincial laboratory information system to the BCCR. Pathology reports that are out of province or from the cancer screening programs are excluded before sending the data to eMaRC. The dotted box on the right shows the manual processing of the reports by the tumor registrars, and the dotted box on the left shows the change in the process when the NLP pipeline is introduced. BCCR, British Columbia Cancer Registry; eMaRC, Electronic Mapping, Reporting, and Coding; HL7, Health Level Seven; LIS, laboratory information system; NLP, natural language processing.

As of 2020, the BCCR receives all provincial pathology reports as HL7 messages in real time from regional laboratory information systems, including cancer and noncancer reports (Fig 2; N = 855,000 reports per year). HL7 is a messaging standard^18^ that defines the structure and content of messages that are exchanged between health care systems. Figure 2 shows the flow of provincial HL7 messages to the BCCR. The BCCR applies exclusions to incoming messages (preprocessing; approximately 285,000 report exclusions per year) including out-of-province residents, invalid messages, and various messages from the cancer screening programs on premalignant lesions.

The remaining messages (570,000 per year approximately) are sent for processing to eMaRC.

### eMaRC

eMaRC^8^ is a software package developed by the US Centers for Disease Control and Prevention for cancer registries. BCCR uses eMaRC (version 6.0.0.5) to distinguish between reportable tumors, nonreportable tumors, and noncancer pathology. The eMaRC algorithm searches a terms table to find potential reportable tumors including terms on the anatomic site, histology, behavior, grade, and laterality.^8^

eMaRC uses a rule-based text analytics engine, which, when optimized to decrease false negatives (*<*1%), results in a large number of false positives (30%-35%). That is, eMaRC classifies many reports as reportable cancer although they are nonreportable. Tumor registrars at the BCCR manually review all reports that eMaRC considers reportable (approximately 127,000 per year) to identify false positives (approximately 38,000 per year; Fig 2).

### Data Set Description

This section provides details on the BCCR pathology report data set used for training and evaluating our proposed method.

#### Training Data

Our training data set consists of 40,000 randomly sampled post-eMaRC pathology reports (step 3 of Fig 2) from 2021, that is, the reports that eMaRC identified as reportable tumors. Among the eMaRC-identified reportables, the prevalence of false positives is 30%-35%, that is, 65%-70% of the reports are true reportables. This distribution is reflected in our training and test data splits.

#### Test Data

A random sample of 10,000 pathology reports from 2021, not included in the training data, was used as the initial test set. To test our proposed model's performance on a diverse set of data, we further used 20,000 reports from 2022 and 400 reports from 2023. Prevalence of true reportables in the test data from 2021, 2022, and 2023 was 35%, 36%, and 37%, respectively. Only 400 reports were chosen from the latest year because these data are not yet processed and required additional, ad hoc manual annotation efforts.

### Models

We use two transformer^9^-based models for our ensemble. Both models are pretrained using clinical documents.

#### The Pretrained Models

1. GatorTron: GatorTron^16,19^ is a large clinical LM, trained using clinical notes from the University of Florida health system covering more than 2 million patients.
2. BlueBERT: BlueBERT^15^ is a BERT-based model pretrained on PubMed abstracts and clinical notes.

#### Fine-Tuning

We fine-tune the pretrained models on the training data (Section Data Set Description) using a desktop machine, with Intel Xeon CPU, 64 GB RAM, and a single NVIDIA RTX A5000 GPU (24 GB VRAM). Our fine-tuning hyperparameters are based on the best practices from the literature to avoid overfitting, such as limiting the number of training epochs,^20^ and are provided in the Data Supplement (Table S1). After fine-tuning on BCCR's data, we denote the models as BCCR-tron and BCCR-BERT, respectively (Contact the author regarding access to models and code). In addition to the different underlying model properties and different fine-tuning hyperparameters, we use different text preprocessing methods to ensure model output diversity for an efficient ensemble. We detail the text preprocessing next.

#### Text Preprocessing

To ensure variability in the model output, we use different text preprocessing techniques for both models, where the input for fine-tuning GatorTron to BCCR-tron consists of removing special characters, removing the standard header that contains identifiable information, and looking for the diagnosis section within the report using regular expressions. If found, we take the first 512 tokens (Tokens are the individual words/subwords from the text that serve as the input units for the model) from the section. If the section is not found, we use the initial 512 tokens. For fine-tuning BlueBERT to BCCR-BERT, we first fine-tune a longformer^21^ based model to extract multiple relevant sections in the report. Once identified, we use the first 512 tokens of the relevant section as an input for fine-tuning BCCR-BERT. Section relevance is based on a hierarchy imposed by subject matter experts, following the same decision-making process as is used during the manual coding process. We provide further details on text preprocessing and segmentation in the Data Supplement.

#### Combining Outputs

We use the OR logic to combine the model outputs. That is, our ensemble outputs Reportable if any of the models' output was Reportable; otherwise, it outputs Non-reportable. Table 1 enumerates the output combinations. This logic ensures that we meet BCCR's target for reportable pathology accuracy.

**TABLE 1.** Combining the Output From Both Models

BCCR-tron	BCCR-BERT	Combined
Reportable	Reportable	Reportable
Nonreportable	Reportable	Reportable
Reportable	Nonreportable	Reportable
Nonreportable	Nonreportable	Nonreportable

Abbreviation: BCCR, British Columbia Cancer Registry.

## RESULTS

This section provides results evaluating our proposed ensemble approach. We compare it with the use of individual models (Section Impact of the Combined Approach), showcase the importance of fine-tuning the pretrained models (Section Impact of Fine-Tuning), and investigate our proposed approach with respect to fairness (Section Investigating Fairness).

BCCR's operational requirement is to capture 98% of the true reportable tumor pathology reports of all true reportable pathology reports (Operational requirement is based on the accuracy of tumor registrars), which is analogous to recall and is defined as follows:

$$\frac{\text{TP}}{\text{TP}+\text{FN}},$$

where TP and FN are true positives and false negatives, respectively. We use this performance metric to report results per-class, and we call it per-class accuracy. For completeness, we supplement the results with precision in the Data Supplement (Table S4).

### Impact of the Combined Approach

We begin our empirical evaluation by evaluating the proposed combined approach (using the OR logic). We compare it with the performance of individual models to study the effectiveness of the combined approach.

Table 2 shows the results by year. BCCR-tron and BCCR-BERT are the models fine-tuned on BCCR pathology reports, and combined is the result from combining both models as explained in Section Combining Outputs. Our proposed combined approach outperforms the individual models for reportable accuracy, achieving a 1%-2% performance gain. This comes at the expense of reduced nonreportable accuracy, which we would expect from using the OR logic. The results are within the prescribed benchmarks and are acceptable for a system to be used in production.

**TABLE 2.** Individual Fine-Tuned Models Versus Combined Output

Year	BCCR-tron	BCCR-BERT	Combined
2021	0.92-0.97	0.94-0.96	0.90-0.98
2022	0.91-0.97	0.94-0.95	0.89-0.98
2023	0.97-0.96	0.95-0.96	0.95-0.98

NOTE. The table shows per-class accuracy, where (left) the first numbers are for nonreportable accuracy and (right) the second numbers are for reportable accuracy. We observe that the combined approach boosts reportable accuracy at the expense of acceptable loss in nonreportable accuracy.

Abbreviation: BCCR, British Columbia Cancer Registry.

As discussed in Section Text Preprocessing, the input to both models varies, where BCCR-BERT uses a curated input using a longformer. However, there is little difference in the performance of either model because the pertinent information required for a report to be classified as reportable versus nonreportable is typically present at the beginning of the report, which is included in the input for BCCR-tron.

The combined approach has been operational at BCCR since January 2024, with results in production mimicking the performance observed during development.

### Impact of Fine-Tuning

This section evaluates the impact of fine-tuning pretrained models using problem-specific data, even when the models are pretrained on domain-specific text.

We observe that the pretrained models without fine-tuning (GatorTron and BlueBERT) perform poorly as zero-shot classifiers (Table 3), whereas their fine-tuned versions (BCCR-tron and BCCRBERT) perform significantly better. These results prove that fine-tuning is crucial, even when the pretrained models are trained on data from similar domains.

**TABLE 3.** Impact of Fine-Tuning

Year	GatorTron	BlueBERT	BCCR-tron	BCCR-BERT
2021	0.99-0.02	0.71-0.35	0.92-0.97	0.94-0.96
2022	0.10-0.86	0.72-0.27	0.91-0.97	0.94-0.95
2023	0.65-0.48	0.50-0.40	0.97-0.96	0.95-0.96

NOTE. The table shows per-class accuracy, where (left) the first numbers are for nonreportable accuracy and (right) the second numbers are for reportable accuracy. We observe that the fine-tuned versions perform significantly better than the pretrained versions.

Abbreviation: BCCR, British Columbia Cancer Registry.

### Investigating Fairness

It is important to measure the possible sources of bias or unfairness when developing and deploying NLP-based methods in high-stake domains such as health care. The unintentional/inbuilt bias in LMs, whether it is a product of training data or training methods, is a significant concern and an active research area.^22,23^ Fairness is defined as the consistent model behavior across different subgroups within the data set, whether the subgroups are based on sex, ethnicity, and so on. An unfair model would prefer one subgroup over the other(s).

For our evaluation, we focus on sex and ethnicity. We consider a model to be fair if it performs similarly well for both sexes and all ethnicities. While sex is available as part of an individual's medical record, ethnicity is not a recorded variable. We use an open-source predictive model, ethnicolr,^24^ to estimate a person's ethnicity given their name. Ethnicolr uses the US census, the Florida voting registration, and Wikipedia data to predict race and ethnicity on the basis of first and last names. This method comes with many caveats regarding underlying biases and assumptions, as noted by the authors,^24^ but it provides us with a starting point to investigate fairness of our proposed method with respect to different population groups. For this evaluation, we combine the test data across 3 years and use the data where ethnicolr can predict the ethnicity, resulting in a total of 11,576 test records.

As the sample size is small for many non-White populations, we combine ethnicity into two groups, White and non-White, resulting in 89% White and 11% non-White population. For a model to be fair, we would expect the model to have similar performance across different ethnicities. Similarly, we would expect the model to perform similarly well for both sexes, where we have 45% males and 55% females.

Table 4 shows that our fine-tuned models and the combined approach perform almost similarly well for both ethnic groups and for both sexes.

**TABLE 4.** Fairness With Respect to Ethnicity and Sex

Group	BCCR-tron	BCCR-BERT	Combined
Ethnicity—White	0.93-0.96	0.95-0.96	0.91-0.98
Ethnicity—non-White	0.92-0.96	0.96-0.95	0.92-0.98
Sex—female	0.93-0.97	0.95-0.96	0.92-0.98
Sex—male	0.93-0.96	0.94-0.95	0.90-0.97

NOTE. We observe that our proposed method performs similarly well for both ethnic groups and for both sexes.

Abbreviation: BCCR, British Columbia Cancer Registry.

## DISCUSSION

To our knowledge, this is the first study to use transformer-based LMs for detection of reportable tumors within a PBCR. We demonstrate our ensemble model approach on unseen pathology reports from different time periods that were considered reportable using current state-of-the-art rule-based text analytics software. However, in reality, these reports represented both reportable and nonreportable tumors. Our proposed approach achieved average accuracies of 98% on reportable tumors and 91% on nonreportable tumors, where the accuracy for reportable tumors is well within the benchmarks for an operational automated system (on the basis of comparison with the accuracy of tumor registrars [Section Results]). We also investigated our models for fairness with respect to patient's ethnicity and sex. Our proposed method provides similar results across different population groups.

Multiple NLP-based approaches have been applied to cancer pathology. A recent systemic review^5^ divides the approaches into three categories: (1) rule-based, (2) non–deep learning-based, and (3) deep learning–based.

Rule-based NLP approaches are commonplace in cancer surveillance and rely on expert-developed rules to extract information from unstructured text.^5,8,11,25^ Significant manual effort to design rules and sensitivity to small changes in languages makes rule-based systems unsustainable in the real-world setting.^5^ Non–deep learning approaches rely on the presence or frequency of word occurrences. As such, they ignore complex sentence representations.^5^ Capturing sentence representations, and the context within which the sentences occur is critically important for many domains and tasks.

Deep learning demonstrates superior performance compared with rule-based and other machine learning approaches because of its ability to create complex sentence representations.^5^ LMs such as BERT^9^ are capable of learning the context around individual words, outperforming other methods.

While the LMs such as BERT^9^ are domain agnostic by design, a LM created for a specific target domain performs better than models trained on out-of-domain data.^26^

In the biomedical domain, many variants of BERT that are trained on clinical data have been released. Some examples include BioBert,^14^ ClinicalBert,^27^ PubMedBert,^28^ PathologyBERT,^5^ CancerBERT,^29^ and so on. A recent addition to pretrained LMs in the health care domain is Path-BigBird,^30^ which is capable of handling longer sequences compared with other models and can classify cancer site, subsite, laterality, histology, and behavior. The pretrained models used in this study, BlueBERT^15^ and Gatortron,^16^ are similarly pretrained on large volumes of clinical text.

Pretrained LMs have significantly advanced NLP research. Practitioners do not need to train models from scratch, which often requires significant computational power and large training data sets. Instead, they can leverage the general learned properties of pretrained LMs, which can be fine-tuned for specific tasks using limited, task-specific data sets. As we have shown in Section Impact of Fine-Tuning, fine-tuning pretrained models on task-specific data boosts their performance significantly compared with using the pretrained models in their zero-shot capacity.

We propose an ensemble-based approach to detect nonreportable tumor pathology reports that were otherwise considered reportable by a rule-based system commonly used among PBCRs in Canada and the United States. The methodology described in this study has significant implications for PBCRs worldwide given that the majority of cancer registries are receiving millions of electronic pathology reports annually directly from laboratory information systems^6^ and must distinguish between reportable and nonreportable cancer. As shown in this study, deep learning methods provide significant advantages over rule-based NLP approaches and can be deployed in cancer surveillance settings to support more timely data collection. Transitioning to deep learning NLP methods represents the next paradigm shift in cancer surveillance.

In the case of the BCCR, the automation of detecting nonreportable tumors enabled the BCCR to save 900 hours of manual work, which is now used for more complex tasks improving timeliness and data quality. Our future work includes investigating the introduction of an LM to act as the preliminary filter to remove the dependence on the rule-based system.

This study has significant strengths. It demonstrates pretrained LMs, fine-tuned on task-specific data and deployed within a cancer registry operational setting, and addresses previous concerns that most studies remain proof-of-concept^5^ and the difficulties of integrating new technology at cancer registries.^6^ In addition, the BCCR is population-based and the data used to develop the approaches were sampled from all provincial cancer pathology reports. This study used open-source models and followed international standards for reportability status (ie, Canadian Cancer Registry guidelines for reportability), enhancing the reproducibility of the approach. Comprehensive evaluation was performed to ensure the reliability of the findings. Furthermore, we evaluated our proposed method for fairness with respect to sex and ethnicity, an important evaluation metric for models deployed in the health care setting. Despite the high accuracy on pathology reports from multiple calendar years, assessment of the generalizability of our proposed ensemble model approach requires external validation on non-BCCR data.

## References

[1] Sung H, Ferlay J, Siegel RL, et al: Global cancer statistics 2020: GLOBOCAN estimates of incidence and mortality worldwide for 36 cancers in 185 countries. CA Cancer J Clin 71:209-249, 2021 33538338 10.3322/caac.21660

[2] Parkin DM: The evolution of the population-based cancer registry. Nat Rev Cancer 6:603-612, 2006 16862191 10.1038/nrc1948

[3] Blumenthal W, Alimi TO, Jones SF, et al: Using informatics to improve cancer surveillance. J Am Med Inform Assoc 27:1488-1495, 2020 32941600 10.1093/jamia/ocaa149 PMC7647312

[4] López-Úbeda P, Martín-Noguerol T, Aneiros-Fernández J, et al: Natural language processing in pathology: Current trends and future insights. Am J Pathol 192:1486-1495, 2022 35985480 10.1016/j.ajpath.2022.07.012

[5] Santos T, Tariq A, Gichoya JW, et al: Automatic classification of cancer pathology reports: A systematic review. J Pathol Inform 13:100003, 2022 35242443 10.1016/j.jpi.2022.100003 PMC8860734

[6] Pollack LA, Jones SF, Blumenthal W, et al: Population heath informatics can advance interoperability: National Program of Cancer Registries electronic pathology reporting project. JCO Clin Cancer Inform 10.1200/CCI.20.00098 10.1200/CCI.20.00098 PMC7608601 33125274

[7] Mapping cancer registry processes to collect and process cancer pathology data in Canada. https://narrative.naaccr.org/article/mapping-cancer-registry-processes-tocollect-and-process-cancer-pathology-data-in-canadacentral-cancer-registriesin-canada/

[8] US Centers for Disease Control and and Prevention: eMaRC plus. https://www.cdc.gov/cancer/npcr/tools/registryplus/mp.htm

[9] Vaswani A, Shazeer N, Parmar N, et al: Attention is all you need. Adv Neural Inf Process Syst 30:1-11, 2017

[10] Alawad M, Gao S, Qiu JX, et al: Automatic extraction of cancer registry reportable information from free-text pathology reports using multitask convolutional neural networks. J Am Med Inform Assoc 27:89-98, 2020 31710668 10.1093/jamia/ocz153 PMC7489089

[11] Hammami L, Paglialonga A, Pruneri G, et al: Automated classification of cancer morphology from Italian pathology reports using natural language processing techniques: A rule-based approach. J Biomed Inform 116:103712, 2021 33609761 10.1016/j.jbi.2021.103712

[12] Gu Y, Tinn R, Cheng H, et al: Domain-specific language model pretraining for biomedical natural language processing. ACM Trans Comput Healthc 3:1-23, 2021

[13] Huang K, Altosaar J, Ranganath R: Clinicalbert: Modeling clinical notes and predicting hospital readmission. arXiv 10.48550/arXiv.1904.05342

[14] Lee J, Yoon W, Kim S, et al: BioBERT: A pre-trained biomedical language representation model for biomedical text mining. Bioinformatics 36:1234-1240, 2020 31501885 10.1093/bioinformatics/btz682 PMC7703786

[15] Peng Y, Yan S, Lu Z: Transfer learning in biomedical natural language processing: An evaluation of BERT and ELMo on ten benchmarking datasets. arXiv 10.48550/arXiv.1906.05474

[16] Yang X, Chen A, PourNejatian N, et al: GatorTron: A large clinical language model to unlock patient information from unstructured electronic health records. arXiv 10.1101/2022.02.27.22271257

[17] Statistics Canada: Surveys and statistical programs—Canadian Cancer Registry (CCR). https://www23.statcan.gc.ca/imdb/p2SV.pl?Function=getSurvey&Id=1517037

[18] Eichelberg M, Aden T, Riesmeier J, et al: A survey and analysis of electronic healthcare record standards. Acm Comput Surv 37:277-315, 2005

[19] Shoeybi M, Patwary M, Puri R, et al: Megatron-lm: Training multi-billion parameter language models using model parallelism. arXiv 10.48550/arXiv.1909.08053

[20] Devlin J, Chang MW, Lee K, et al: Bert: Pre-training of deep bidirectional transformers for language understanding. arXiv 10.48550/arXiv.1810.04805

[21] Beltagy I, Peters ME, Cohan A: Longformer: The long-document transformer. arXiv 10.48550/arXiv.2004.05150

[22] Dixon L, Li J, Sorensen J, et al: Measuring and mitigating unintended bias in text classification. Proceedings of the 2018 AAAI/ACM Conference on AI, Ethics, and Society, New Orleans, LA, February 1-3, 2018

[23] Stanovsky G, Smith NA, Zettlemoyer L: Evaluating gender bias in machine translation. arXiv 10.48550/arXiv.1906.00591

[24] Chintalapati R, Laohaprapanon S, Sood G: Predicting race and ethnicity from the sequence of characters in a name. arXiv 10.48550/arXiv.1805.02109

[25] Osborne JD, Wyatt M, Westfall AO, et al: Efficient identification of nationally mandated reportable cancer cases using natural language processing and machine learning. J Am Med Inform Assoc 23:1077-1084, 2016 27026618 10.1093/jamia/ocw006 PMC5070519

[26] Raffel C, Shazeer N, Roberts A, et al: Exploring the limits of transfer learning with a unified text-to-text transformer. J Mach Learn Res 21:1-67, 202034305477

[27] Alsentzer E, Murphy JR, Boag W, et al: Publicly available clinical BERT embeddings. arXiv 10.48550/arXiv.1904.03323

[28] Gu Y, Tinn R, Cheng H, et al: Domain-specific language model pretraining for biomedical natural language processing. arXiv 10.48550/arXiv.2007.15779

[29] Zhou S, Wang N, Wang L, et al: CancerBERT: A cancer domain-specific language model for extracting breast cancer phenotypes from electronic health records. J Am Med Inform Assoc 29:1208-1216, 2022 35333345 10.1093/jamia/ocac040 PMC9196678

[30] Chandrashekar M, Lyngaas I, Hanson HA, et al: Path-BigBird: An AI-driven transformer approach to classification of cancer pathology reports. JCO Clin Cancer Inform 10.1200/CCI.23.00148 10.1200/CCI.23.00148 PMC10904099 38412383

